# The chloroplast genome of an important herb species, *Ephedra sinica* (Ephedraceae)

**DOI:** 10.1080/23802359.2019.1687355

**Published:** 2019-11-08

**Authors:** Yifeng Lin, Tianyi Cao, Quan Zhang

**Affiliations:** a The Second Affiliated Hospital of Wenzhou Medical University, Wenzhou, China;; b Zhejiang Chinese Medical University, Hangzhou, China;; c Hangzhou Red Cross Hospital, Hangzhou, China

**Keywords:** *Ephedra sinica*, Ephedraceae, chloroplast genome, phylogenetic analysis

## Abstract

*Ephedra sinica* has been utilized by humans for over 5000 years as Chinese herbal medicine in China. In this study, we reported the complete chloroplast genome of *E. sinica*. The chloroplast genome of *E. sinica* is 109,550 bp in length as the circular, which harbors a large single-copy (LSC) region of 59,962 bp, a small single-copy (SSC) region of 8106 bp and separated by a pair of inverted-repeat (IR) regions of 20,742 bp each. The overall nucleotide content of the chloroplast genome: A of 31.2%, T of 32.1%, C of 18.4% G of 18.3%, and 36.7% GC content. This chloroplast genome contains 118 genes, which includes 74 protein-coding genes (PCGs), 36 transfer RNA (tRNAs) and 8 ribosome RNA (rRNAs). Phylogenetic analysis result showed that *E. sinica* was most closely related to *Ephedra equisetina* in the family Ephedraceae using the Neighbor-Joining (NJ) method.

*Ephedra sinica* (Mahuang) is a medicinal preparation from the plant *E. sinica*. Several additional species belonging to the genus Ephedra have traditionally been used for a variety of medicinal purposes, which has been used in traditional Chinese medicine herb for more than 5000 years (Yoshizawa et al. 2005). The stems of most members in the family Ephedraceae contain the alkaloid ephedrine, which is used for treatment of common cold, treatment of asthma and other respiratory ailments (Hong et al. 2011). About, little information is known about the genome of *E. sinica*. So, in this study, the chloroplast genome of *E. sinica* was assembled, annotated and reported, which can provide genomic information and data for the family Ephedraceae plant in further, also will be important to research and development of drugs for treatment of cold and asthma.

The fresh stem sample of *E. sinica* was collected from Chinese medicine market near Zhejiang Chinese Medical University (Hangzhou, Zhejiang, China, 30.09 N, 119.89E). The chloroplast genomic DNA of *E. sinica* was extracted from the fresh stem using the modified CTAB method and stored in Zhejiang Chinese Medical University (No. SCMC-ZJU-TCM-02). The chloroplast DNA was purified and fragmented using the NEB Next Ultra^TM^ II DNA Library Prep Kit (NEB, BJ, and CN) that was sequenced and analyzed. FastQC software (Andrews 2015) was used to perform and remove low-quality reads and adapters for quality control. The chloroplast genome was assembled and annotated using the MitoZ software (Meng et al. 2019). OrganellarGenomeDRAW web (Greiner et al. 2019) was used to draw the physical map of the chloroplast genome of *E. sinica*.

The chloroplast genome of *E. sinica* is 109,550 bp in length which is the circular with the overall nucleotide content of the chloroplast genome: 31.2% A (Adenine), 32.1% T (Thymine), 18.4% C (Cytosine), 18.3% G (Guanine), and 36.7% GC content. It harbors a characteristic quadripartite structure with a large single-copy (LSC) region of 59,962 bp, a small single-copy (SSC) region of 8106 bp and separated by a pair of inverted repeat (IR) regions of 20,742 bp each. The chloroplast genome of *E. sinica* contains 118 genes, which includes 74 protein-coding genes (PCGs), 36 transfer RNA genes (tRNAs) and 8 ribosomal RNA genes (rRNAs). 18 genes were found duplicated in each IR regions, which included 6 PCG genes species (*ycf2*, *rps7*, *rps12*, *chlL*, *chlN a*nd *rps15*), 8 tRNA genes species (*trnH-GUG*, *trnI-CAU*, *trnL-CAA*, *trnV-GAC*, *trnI-GAU*, *trnA-UGC*, *trnR-ACG* and *trnN-GUU*) and 4 rRNA genes species (*rrn16, rrn23, rrn4.5* and *rrn5*). This chloroplast genome has submitted to the GenBank and NCBI accession No.MK9677872.

To further investigate *E. sinica* phylogenetic position, the Neighbor-Joining (NJ) method was used for construct phylogenetic tree. NJ phylogenetic tree analysis used MEGA X (Kumar et al. 2018) and performed using 2000 bootstrap values replicate at each node. All of the nodes were inferred with strong support by the NJ methods. The final NJ phylogenetic tree was edited using the iTOL version 4.0 (https://itol.embl.de/) (Letunic and Bork 2016). The NJ phylogenetic tree (Figure 1) showed that *E. sinica* was most closely related to *Ephedra equisetina* (MH161420.1). This study, is very important for research and development of drugs for treatment of cold and asthma in futher.

**Figure 1.**
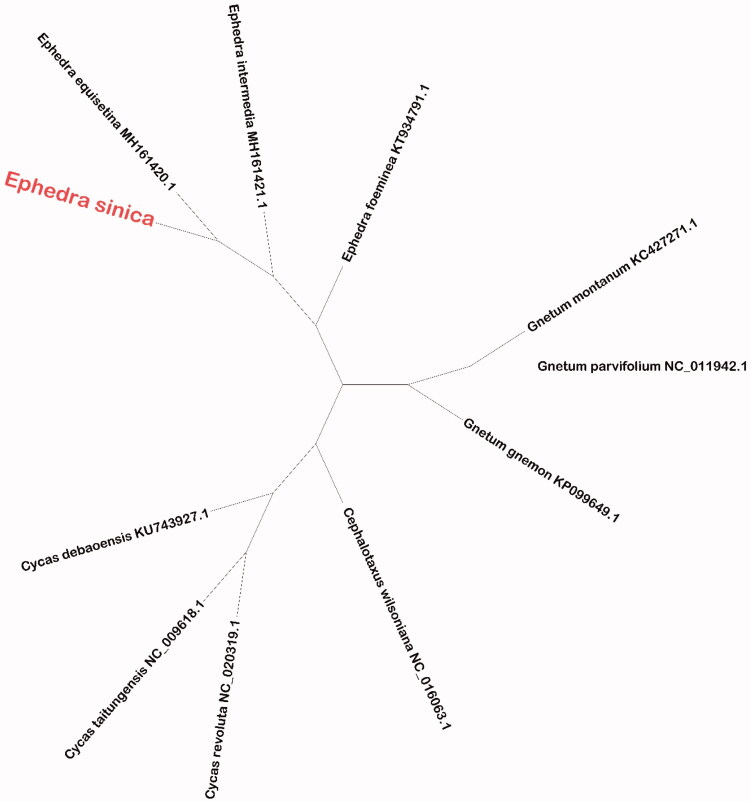
Phylogenetic relationships of *Ephedra sinica* with other 10 the family Ephedraceae species chloroplast genome sequences uesd the Neighbor-Joining (NJ) method analysis. Bootstrap support values based on 2000 replicates are shown next to the nodes for each branch.
